# Evaluating the aerobic xylene-degrading potential of the intrinsic microbial community of a legacy BTEX-contaminated aquifer by enrichment culturing coupled with multi-omics analysis: uncovering the role of *Hydrogenophaga* strains in xylene degradation

**DOI:** 10.1007/s11356-021-18300-w

**Published:** 2022-01-06

**Authors:** Sinchan Banerjee, Anna Bedics, Péter Harkai, Balázs Kriszt, Nagaraju Alpula, András Táncsics

**Affiliations:** 1 Department of Molecular Ecology, Institute of Aquaculture and Environmental Safety, Hungarian University of Agriculture and Life Sciences, Gödöllő, Hungary; 2 Department of Environmental Safety, Institute of Aquaculture and Environmental Safety, Hungarian University of Agriculture and Life Sciences, Gödöllő, Hungary; 3 Department of Biotechnology, Microbial Biotechnology Research Unit, Kakatiya University, Warangal, India

**Keywords:** Xylene, *Hydrogenohaga*, Groundwater, Aromatic hydrocarbons, Biodegradation, Catechol 2,3-dioxygenase

## Abstract

To develop effective bioremediation strategies, it is always important to explore autochthonous microbial community diversity using substrate-specific enrichment. The primary objective of this present study was to reveal the diversity of aerobic xylene-degrading bacteria at a legacy BTEX-contaminated site where xylene is the predominant contaminant, as well as to identify potential indigenous strains that could effectively degrade xylenes, in order to better understand the underlying facts about xylene degradation using a multi-omics approach. Henceforward, parallel aerobic microcosms were set up using different xylene isomers as the sole carbon source to investigate evolved bacterial communities using both culture-dependent and independent methods. Research outcome showed that the autochthonous community of this legacy BTEX-contaminated site has the capability to remove all of the xylene isomers from the environment aerobically employing different bacterial groups for different xylene isomers. Interestingly, polyphasic analysis of the enrichments disclose that the community composition of the *o*-xylene-degrading enrichment community was utterly distinct from that of the *m*- and *p*-xylene-degrading enrichments. Although in each of the enrichments *Pseudomonas* and *Acidovorax* were the dominant genera, in the case of *o*-xylene-degrading enrichment *Rhodococcus* was the main player. Among the isolates, two *Hydogenophaga* strains, belonging to the same genomic species, were obtained from *p*-xylene-degrading enrichment, substantially able to degrade aromatic hydrocarbons including xylene isomers aerobically. Comparative whole-genome analysis of the strains revealed different genomic adaptations to aromatic hydrocarbon degradation, providing an explanation on their different xylene isomer-degrading abilities.

**Supplementary Information:**

The online version contains supplementary material available at 10.1007/s11356-021-18300-w.

## Introduction

Xylene is one of the volatile organic compounds composed of a central benzene ring with two methyl groups attached as substituents, arranged in three various positions to create three distinct isomers: *m*-xylene, *o*-xylene, and *p*-xylene (Marshall and Rodgers 2008; Kandyala et al. 2010; Yan and Zhou 2011). It is a toxic monoaromatic hydrocarbon and is highly mobile in the environment, either in the gaseous, liquid, or solid phase (Mazzeo et al. 2013). Among BTEX compounds, xylene is most commonly used as a solvent in industrial coatings and the petrochemical industry (Boonsaner et al. 2011). Only in the USA, the amount of xylenes (mixed isomers) released in 2012 from all factories was 7,482,435 kg (including disposal and release on-site and off-site) (US EPA-United States Environmental Protection Agency 2012). It could also be released into nature because of crude oil spillages during shipping, loading, and storage. As a result of these different practices and procedures, the deposition of xylene in soil and groundwater can pose a tremendous challenge to soil and groundwater resources (Kao and Wang 2000; Kandyala et al. 2010; Atlas and Hazen 2011; Das and Chandran 2011; Alrumman et al. 2015). Because of their relatively high water solubility, this type of contamination was often treated as a considerable threat to the primary drinking water reserves. Since xylene has been reported as toxic to the liver, kidneys, and central nervous system, once it enters into the body by skin contact or inhalation, hence, monitoring and removal of xylene from the environment have been regarded globally as a high priority (Kao and Wang 2000; Andreoni and Gianfreda 2007; Kandyala et al. 2010; Atlas and Hazen 2011; Das and Chandran 2011).

Although eradication of xylene and other petroleum hydrocarbons can be performed using physical, chemical, and biological approaches, microbial biodegradation is considered less expensive and one of the most promising alternatives means for cleaning up the environment from petroleum hydrocarbon contamination (Kovalick 1992; Vidali 2001; Jahn et al. 2005; Vieira et al. 2007; Silva et al. 2009; Lin et al. 2010; Das and Chandran 2011; Alrumman et al. 2015).

The technique of biodegradation uses microorganisms to treat contaminants by degrading organic compounds into less harmful material, such as CO_2_, methane, water, and inorganic salts, under both *ex-situ* or *in-situ*, aerobic or anaerobic conditions. BTEX biodegradation using microorganisms has also been established as an effective and environmentally friendly method (Delhoménie et al. 2003; Noguchi et al. 2014; Kim et al. 2014). There are several studies conducted that found the role of various microorganisms for effective degradation of xylene, including *Alcaligenes xylosoxidans*, *Pseudomonas putida* (Reardon et al. 2000; Yeom and Daugulis 2001; Robledo-Ortíz et al. 2011), *Pandoraea* sp. (Wang et al. 2015), and *Rhodococcus* sp., (Di Canito et al. 2018). A few isolates were reported to grow with *m*-xylene (Dolfing et al. 1990; Fries et al. 1994; Rabus and Widdel 1995; Harms et al. 1999; Morasch et al. 2004) or *o*-xylene (Harms et al. 1999; Morasch et al. 2004), whereas until now, only handful of studies have reported on pure cultures, which can degrade *p*-xylene anaerobically (Higashioka et al. 2012). So far, limited number of studies have been undertaken regarding xylene degradation by the autochthonous microbial community from decade-old xylene-contaminated site to develop effective strategies for bioremediation using biostimulation. To add further, not much have been investigated concerning the xylene biodegradation potential of the genus *Hydrogenophaga*, in spite of their noticeable presence in xylene-contaminated environments (Táncsics et al. 2010, 2012, 2013; Benedek et al. 2016). In the aerobic degradation of aromatic hydrocarbons ring-cleavage dioxygenases are playing a key role, by catalyzing aromatic ring-opening reactions. It is well known that in contaminated subsurface environments subfamily I.2.C-type catechol 2,3-dioxygenase (*C23O*) genes are usually present in large diversity (Táncsics et al. 2013). Most of them are harbored by members of the *Betaproteobacteriales* (Táncsics et al. 2013) and some of them are involved in the microaerobic degradation of aromatics, e.g., toluene (Táncsics et al. 2020). Still, the role of both of these bacteria and the I.2.C-type *C23O* genes in xylene degradation is unknown. Despite several studies, still, there are underlying information that need thorough investigation to uncover.

These aforementioned facts prompted us to explore structural changes in the autochthonous microbial communities in xylene isomer induced enrichments and uncover information about key indigenous degraders of a decade-old petroleum-contaminated aquifer. Nevertheless, this present study is the first one of its kind, where we furnished information about critical community shift induced by xylene isomers as sole carbon source and the detailed study of xylene degradation by *Hydrogenophaga* strains. Furthermore, comparative analysis of the whole-genome sequence of two *Hydrogenophaga* strains that belong to the same species with entirely different substrate utilization patterns shed light on the community-acquired functional capabilities.

## Materials and methods

### Site description and sample collection

Groundwater sample was obtained from a gasoline-contaminated site of Siklos, a town located in the south-west region of Hungary (45′51″25.8°N, 18′17″32.3°E) (Táncsics et al. 2013) during February 2019. This site was contaminated by petroleum hydrocarbons due to accidental leakage of the underground storage tank of a former petrol station during the end of 1980s, resulting in hydrocarbon contamination of surrounding soil and groundwater reserve. Groundwater sample was collected from the monitoring well No. ST2 located at the center of the contaminated zone from a depth of ~ 2.5–4 m. The sampled groundwater is mainly contaminated with xylene, benzene, ethylbenzene, and toluene. The site has been thoroughly studied and described by Táncsics et al. (2012, 2013) earlier. Physicochemical properties and BTEX concentration of contaminated groundwater are monitored orderly by an accredited laboratory (Wessling Hungary Ltd.) according to Hungarian Standard (MSZ) analytical techniques to enable the authorities to plan for strategic measures. The dissolved oxygen concentration and redox potential values were measured on-site using a WTW hand-held Meter Multi 350i (WTW, Germany). The sample was collected into sterile 1-L serum bottles, keeping no headspace, and transported to the laboratory, preserved at 4 °C in the dark for further investigation. Physicochemical characteristics of the sample are summarized in Table S1.

### Enrichment setup and evaluation of xylene degradation

Enrichment microcosms were set up in duplicates (2 parallel) with contaminated groundwater sample from the Siklos site using *m*-, *p*-, and *o*-xylene as a sole carbon source under aerobic conditions (~ 7–8 mg L^−1^ dissolved O_2_). For the enrichments, 45 mL minimal mineral salt (MS) medium (Fahy et al. 2006) supplemented with vitamins (1 mg/L thiamine, 15 μg/L biotin, and 20 μg/L vitamin B_12_) was used in 100-mL serum bottles sealed with butyl-rubber septa and aluminium crimp seals. Subsequently, *m*-, *p*-, or *o*-xylene as sole carbon and energy sources were added to the enrichments at a final concentration of 1 mM followed by inoculation with 5 mL of the contaminated groundwater sample. To ensure the strict aerobic conditions, the bottles were monitored non-invasively by Fibox 3 trace v3 fiber optic oxygen meter with PSt3 sensor spots (PreSens). The oxygen concentration was restored whenever needed by injecting sterile air (0.2-μm pore size filtered) through the butyl-rubber septa. The microcosms were incubated for 7 days in a shaking thermostat at 28 °C, 150 rpm. Then 5-mL inoculum from each microcosm was transferred into a fresh medium. Similar transfers were made for consecutive 5 weeks. To monitor the consumption of xylene as the sole carbon source, the concentration of *m-*, *p-*, and *o*-xylene was measured for 5th-week enrichment microcosms at 24-h interval by headspace analysis on an ISQ Single Quadrupole gas chromatography-mass spectrometer (GC–MS) (Thermo Fischer Scientific) via a SLB-5 ms fused silica capillary column (Supelco Analytical). Uninoculated duplicate microcosms with 45 mL of medium and 1 mM concentration of *m-*, *p-*, and *o*-xylene served as a negative control for GC–MS analysis. For the analysis, the oven temperature was set to 40 °C for 3 min, then ramped at a rate of 20 °C min^−1^ to 190 °C, and held for 1 min. The mass spectrometer ran at 250 °C in full scan mode.

### Community DNA isolation and T-RFLP fingerprinting

Genomic DNA from the enrichments was isolated after five consecutive transfers. In order to do so, microbial biomasses were harvested from 45 mL of the enrichment by centrifuging at 2360 g at 4 °C for 10 min using a Rotanta 460 R (Hettich). According to the manufacturer’s instructions, total community DNA was extracted from the pellet using the DNeasy Ultraclean Microbial Kit (Qiagen). Followed by isolation, the quality of DNA was analyzed in ethidium bromide stained 1% (w/v) agarose gel electrophoresis. Moreover, the concentration of extracted DNA was measured using a NanoDrop One Microvolume UV–Vis spectrophotometer (Thermo Fisher Scientific, Waltham, MA, USA). To get a comprehensive view concerning the differences in the community structure in various enrichments and to decide on the enrichment sample that represents the phylogenetic diversity best among duplicates, for further 16S rRNA gene Illumina sequencing, terminal restriction fragment length polymorphism (T-RFLP) analysis was performed (Székely et al. 2009). For 16S rDNA-based community profile study, the VIC-labeled amplicons were generated using 27f-VIC (5′-AGAGTTTGATCMTGGCTCAG-3′) and 1492r primers (5′T ACGGYTACCTT GTTACGA C T T-3′). Amplification conditions were the same as described earlier by Benedek et al. (2016). All amplifications were achieved in a ProFlex PCR System (Life Technologies). The quality of amplified products was checked by electrophoresis on 1% agarose gels stained with ethidium bromide. The VIC-labeled 16S rDNA were digested with 1U *Rsa*I (GT↓AC) (Thermo Fisher Scientific) for 1.5 h at 37 °C. The generated fluorescently labeled terminal restriction fragments (T-RFs) were purified by the ethanol precipitation method. After ethanol precipitation, fragments were separated on a Model 3130 Genetic Analyzer (Applied Biosystems), while using GeneMapper 4.0 software (Applied Biosystems), preliminary evaluation of electropherograms was performed. T-RFLP data were handled as described earlier (Farkas et al. 2017). Statistical analysis, such as cluster analysis (Bray–Curtis method) of the T-RFLP electropherograms, was performed using the PAST 3.26 software package (Hammer et al. 2001).

### Illumina 16S rRNA gene amplicon sequencing for community analysis

Using the method of T-RFLP, one of the representatives among the duplicate enrichments were selected (M1, P1, O1) for amplicon sequencing for in-depth evaluation of community composition. The V3 and V4 variable regions of the 16S rRNA gene were amplified with the PCR primers suggested by Klindworth et al. (2013) to get paired-end 16S rDNA amplicons. PCR was done according to the 16S metagenomic sequencing library preparation guide of Illumina using KAPA HiFi HotStart Ready Mix-et (KAPA Biosystems). Paired-end fragment reads were generated on an Illumina MiSeq sequencer using MiSeq Reagent Kit v3 (600-cycle) by SeqOmics Biotechnology Ltd. (Mórahalom, Hungary). Read numbers were as follows: 235,746 for enrichment M1, 246,389 for enrichment P1, and 245,628 for enrichment O1. Primary data analysis (base-calling) was conducted with Bbcl2fastq^˄^ software (v2.17.1.14, Illumina). Reads were quality and length trimmed in CLC Genomics Workbench Tool 9.5.1 using an error probability of 0.05 (Q13) and a minimum length of 50 nucleotides as a threshold. Then the rimmed sequences were processed using mothur v1.41.1 (Schloss et al. 2009) as recommended by the MiSeq SOP page (http://www.mothur.org/wiki/MiSeq_SOP) (Kozich et al. 2013). Based on the alignment using the SILVA 132 SSURef NR99 database (Quast et al. 2013), sequences were assorted. Detection of chimeras was done with Mothur’s uchime command (Edgar et al. 2011), and the “split.abund” command was also used to remove singleton reads according to Kunin et al. (2010). Ninety-seven-percent similarity threshold level was used to assign Operational Taxonomic Units (OTUs) as suggested by Tindall et al. (2010) for prokaryotic species delineation. Venn diagrams were generated by the mothur’s venn command. Rarefaction curves (Fig. S1) showed high sequencing coverage in all samples. Sequence reads were deposited in NCBI under BioProject ID PRJNA704261.

### I﻿dentification of bacterial strains and sequencing of *C230* gene

Cultivable bacterial strains from the xylene-induced enrichments were isolates by serially diluting the homogeneous sample with physiological salt solution (0.9% NaCl). Then 0.1 mL of serially diluted samples was spread on to R2A agar plates (protease peptone 0.5 g, casamino acids 0.5 g, yeast extract 0.5 g, dextrose 0.5 g, soluble starch 0.5 g, dipotassium phosphate 0.3 g, MgSO_4_ 7H_2_O 0.05 g, sodium pyruvate 0.3 g, agar 15 g, pH 7 ± 0.2) (Sigma-Aldrich, Germany). After 1 week of incubation at 28 °C, then colonies with different morphologies were purified using streak plate technique and maintained on R2A agar slants at 28 °C. Genomic DNA of isolates was extracted from pure cultures grown on R2A agar slants using the UltraClean Microbial DNA Kit (Qiagen) following the manufacturer’s instruction. Afterward, the 16S rRNA and subfamily I.2.C-type *C23O* genes of the isolates were amplified and sequenced. Detection and sequencing of subfamily I.2.C-type *C23O* genes were performed by using the primers XYLE3f (5′-TGY TGG GAY GAR TGG GAY AA-3′) and XYLE3r (5′-TCA SGT RTA SAC ITC SGT RAA-3′) (Táncsics et al. 2012, 2013). The species-level identification of cultivable bacteria was assessed on the basis of 16S rRNA gene-based Sanger dideoxy chain termination sequencing using the universal bacterial primers 27F (5′-AGAGTTTGATC(A/C) TGGCTCAG-3′) and 1492R (5′-TACGG(C/T) TACCTTGTTACGAC TT-3′). Sequencing of the 16S rDNA and *C23O* amplicons was performed according to Benedek et al. (2018).

The 16S rRNA and *C23O* gene sequences of the isolates were deposited to GenBank under the accession numbers MW647763-MW647782, MZ127192, and MW691988–MW691994 subsequently.

### BTEX-degradation analysis of the selected isolates

Aerobic degradation ability of selected strains was accessed by GC–MS. In this study, importance was given to those isolated strains that belong to genera with the least available knowledge regarding their BTEX degradation potential, with particular emphasis on xylene degradation for further investigation. The experiment was conducted in triplicates of 100-mL crimped serum bottles containing 50 mL MS medium supplemented with different BTEX compounds as a carbon source at 5 mg/L concentration and kept aside for 24 h to provide time for the abiotic solution to reach the equilibrium state. Thereafter, investigational 100-µL bacterial inoculum (OD600 0.5) was added to the serum bottles and uninoculated serum bottles served as negative controls. All of the bottles were kept for incubation at 28 °C and 150 r.p.m. in a rotary shaker incubator. Biodegradation of BTEX compounds was measured at a regular interval of 24 h by headspace analysis in GC–MS as described earlier.

### Comparative whole-genome analysis of selected isolates

Despite the fact that genus *Hydrogenophaga* contains excellent BTEX degraders (Fahy et al. 2008; Jechalke et al. 2013), unfortunately, it has not been studied with particular emphasis to our knowledge. Even though previous studies using the same contaminated groundwater sample showed the presence of this genera in different enrichments but never investigated thoroughly. Moreover, there is no such valuable xylene degradation data available to date concerning the isolated *Hydrogenophaga* spp. and their closest relatives. Therefore, to fill that knowledge gap, it was decided to investigate furthermore about the isolated *Hydrogenophaga* strains concerning their aromatic hydrocarbon degradation. The whole-genome sequencing of selected *Hydrogenophaga* strains was performed as described by Borsodi et al. (2019) by the SeqOmics Biotechnology Ltd. (Mórahalom, Hungary). *De novo* assembly and scaffolding was performed with SPAdes version 3.13.0 (Nurk et al. 2013). Automatic annotation of the genome was performed by the NCBI Prokaryotic Genomes Automatic Annotation Pipeline (PGAP) v4.5 (Tatusova et al. 2016) To determine digital DNA–DNA hybridization (dDDH) value between analyzed strains the Genome-to-Genome Distance Calculator (GGDC, https://ggdc.dsmz.de/, version 2.1) was used (Meier-Kolthoff et al. 2013). For the calculation of orthologous average nucleotide identity (OrthoANI) value between strains, the OAT software was used (Lee et al. 2016). To analyze metabolic potentials of the investigated *Hydrogenophaga* strains the genomes were annotated by using the Genoscope platform MAGE (Vallenet et al. 2009) as well. Subsequently, analysis was performed by combining automated annotation from MAGE and manual curation using information from MetaCyc (Caspi et al. 2014), KEGG (Kanehisa and Goto 2000), and UniProt (Bateman 2019). For the alignment of analyzed genomes the CLC Genomics Workbench version 21.0.3 (Qiagen) was used. For the identification of genomic islands the IslandViewer 4 was used (Bertelli et al. 2017).

The genome sequences of strain *Hydrogenophaga* sp. D2P1^T^ and *Hydrogenophaga* sp. D2P3 have been deposited at the GenBank database under the WGS accession numbers VYGV00000000 and JAGPWB000000000.

## Results and discussion

### Physicochemical analysis of investigated groundwater

As the groundwater at the contaminated site is present under a thick layer of clay, the contaminated groundwater well upholds an oxygen-limited setting. Dissolved oxygen concentration values (1.2 mg/L) indicated the same hypothesis. The physicochemical data shown in Table S1 support the statement adequately. Moreover, the data suggest that the reducing condition is prevalent in the contaminated zone. Though the concentration of contaminants significantly decreased compared to the beginning of the contamination with time because of the autochthonous microbial community, the concentrations of some of the contaminants are still beyond the permissible limit according to Hungarian standards. Nowadays, the contaminated aquifer is overdominated with the contamination of xylene. Furthermore, the data shows the decreased concentration of nitrate and sulfate and increasing trend of Fe(II) and methane, which is presumably indicative of the presence of Fe(II) reducing and methanogenic organisms in deeper layers of the contaminated aquifer (Table S1).

### Xylene degradation by enriched communities

**Fig. 1:**
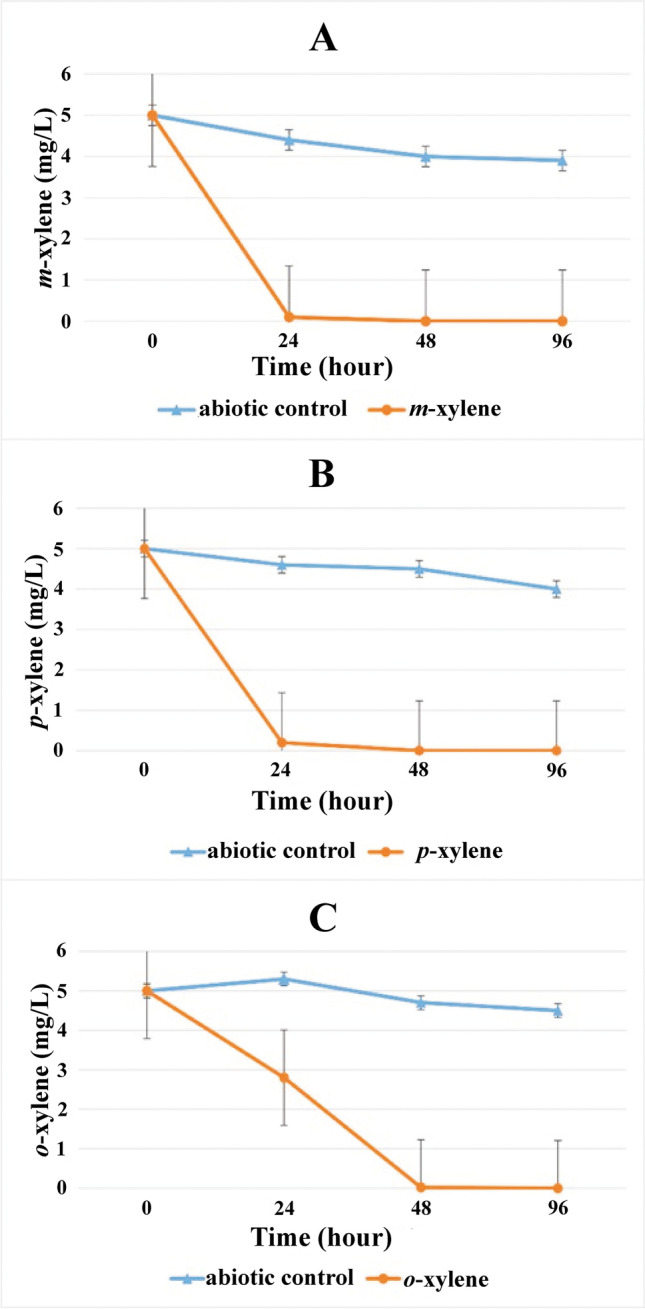
Degradation process of xylene isomers in the aerobic enrichments containing (A) *m*-xylene, (B) *p*-xylene, and (C) *o*-xylene. Xylene concentrations were measured by GC–MS analysis at the 5th week of enrichment as described in the main text. Means of duplicate enrichments are given (with standard error)

Using the contaminated groundwater inoculum, aerobic (7–8 mg/L dissolved oxygen) microcosms were set up in duplicates with *m-*, *p-*, and *o*-xylene as a sole source of carbon and energy. Enrichments were then transferred weekly for consecutive 5 weeks. The xylene degradation process by enrichments reflected that highly competent aerobic-degrading communities evolved in the enrichments by the fifth week. The carbon source in the enrichments was almost depleted by 24 h of incubation; *o*-xylene-degrading enrichments were the only exception (Fig. 1). Among the enrichments, *m*-xylene degradation was the fastest, where enriched community members took 24 h to degrade 5 mg/L concentration of *m*-xylene (Fig. 1, panel A), while *p*-xylene enriched community members took more than 24 h and less than 48 h to degrade 5 mg/L *p*-xylene (Fig. 1, panel B). However, *o*-xylene degradation was a bit slow compared to other enrichments. Community members consumed the same amount of *o*-xylene by 48 h as a carbon source (Fig. 1, panel C). The complete degradation of the added BTEX compounds was confirmed by the change of initial concentration of 5 to 0 mg/L at the end of the experiment.

### Microbial community compositions as revealed by 16S rRNA gene T-RFLP and Illumina 16S rRNA gene amplicon sequencing

Since T-RFLP analysis has been successfully applied to study microbial communities of various microbial ecosystems (Scala and Kerkhof 2000; Schütte et al. 2008), thus as the first step of community composition evaluation, culture-independent 16S rDNA-based T-RFLP analysis was performed using community DNA of the 5th-week enrichments. The primary data of T-RFLP analysis of enrichments and their duplicates revealed the fact about the similarity of the replicates and their community representation.

**Fig. 2:**
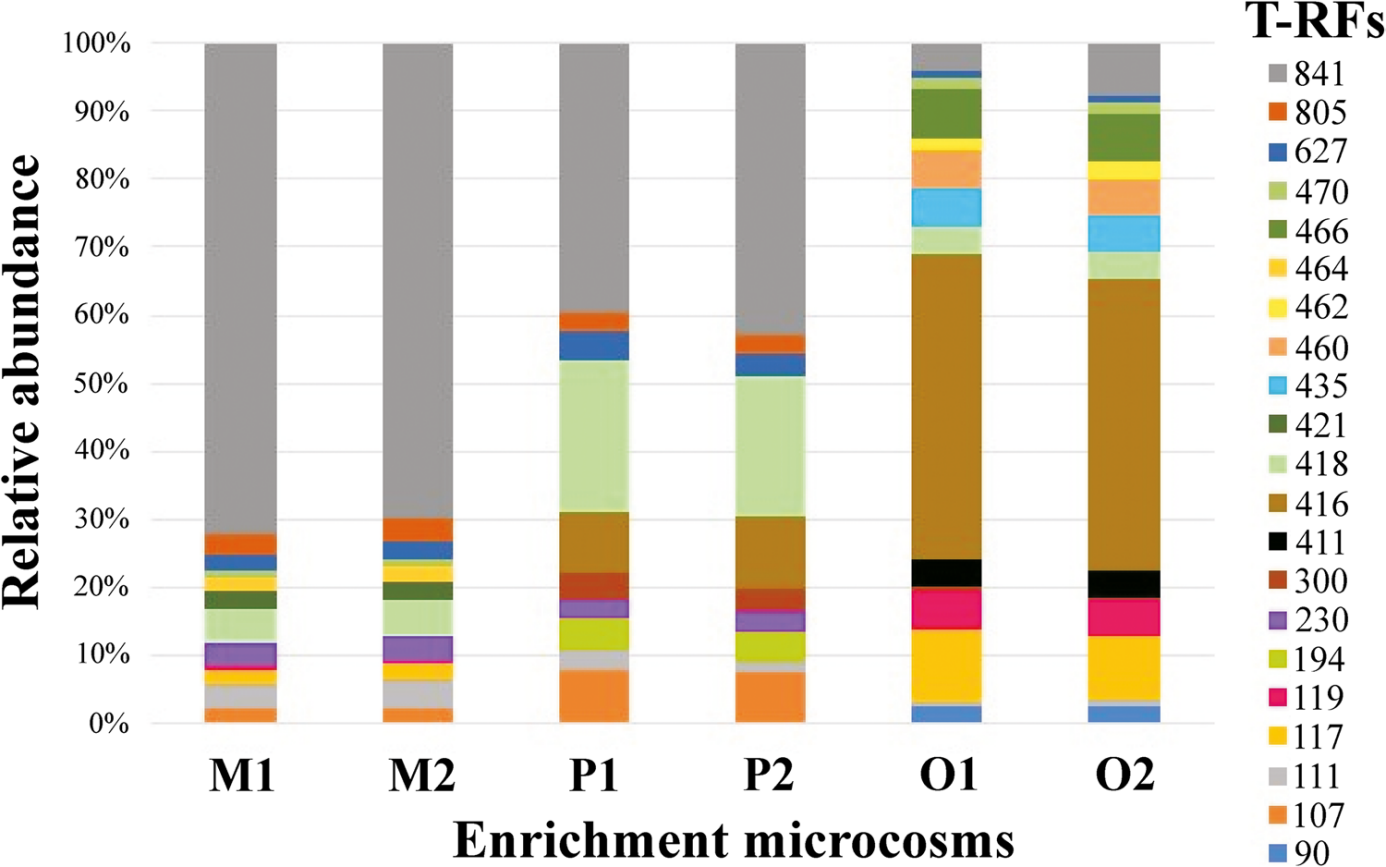
Bacterial community structure of *meta*- (M1, M2), *para*- (P1, P2), and *ortho*-xylene-degrading (O1, O2) enrichment cultures as revealed by 16S rRNA gene-based T-RFLP

The bar plot of T-RFLP fingerprints clearly showed that the composition of the bacterial community of *m-*, *p*-, and *o*-xylene-degrading enrichments were distinctly different, and the community composition was relatively similar for replicates (Fig. 2). The most dominant T-RFs in *m*-xylene-degrading enrichments were 841 bp (approximately 72%) and 418 bp (approximately 5%), in *p*-xylene-degrading enrichments 841 bp (approximately 40%) and 418 bp (20%), while in *o*-xylene-degrading enrichments 117 bp (10%) and 416 bp (44%). The most prominent T-RF that was detectable in all three types of enrichments in different fractions was the 841 bp. Major difference creating T-RFs that differentiated the *o*-xylene-degrading enrichments from *m*- and *p*-xylene-degrading enrichments were 411 bp, 435 bp, 460 bp, and 466 bp. T-RFLP data also suggested that three types of xylene isomer-degrading enrichments differed significantly in the Shannon-H (*H*′), Simpson_1-D, and Chao-1 diversity indexes (Table S2). The highest Shannon (*H*) diversity and Chao-1 values were registered in the *o*-xylene-degrading enrichment samples. Contrarily, the lowest Chao-1 values were recorded in *p*-xylene-degrading enrichments, while the Shannon index was observed as lowest in *m*-xylene-degrading enrichments. Considering these results, it could be presumed that *o*-xylene-degrading enrichment communities were the most diverse and species-rich comparing to the others. The Chao1 index was used to estimate the total number of T-RFs in the community. The Chao1 values of the six samples from high to low were O1 > O2 > M1 > M2 > P1 > P2, which indicated the total number of similar kinds of T-RFs that is indicative of species. The Shannon index ranged from 1.221 to 2.073, and values of the six samples from high to low were O2 > O1 > P1 > P2 > M2 > M1. At the same time, the Simpson index ranged from 0.468 to 0.787. The changes in the value of two indexes showed a similar trend, which indicated that the *m*- and *p*-xylene-degrading enrichment sample in this study contained most of the common T-RF groups. Notably, *o*-xylene-degrading enrichments showed a different trend.

Furthermore, it is interpretable that although *m*-xylene-degrading enrichment showed a higher Chao 1 value still registered a lower Shannon value because the sample had higher species richness but lower evenness. Likewise, the *p*-xylene-degrading enrichment sample had lower species richness but higher evenness. A similar trend could be spotted in the case of Simpson index data also. A higher value of the Simpson index indicates higher diversity. Rare species play a limited role in Simpson’s diversity index, while common species play a more significant role. The lower the value of Shannon’s index is, the lower the diversity is; the lower the value of Simpson’s index (range: 0–1) is, the higher the diversity is.

**Fig. 3:**
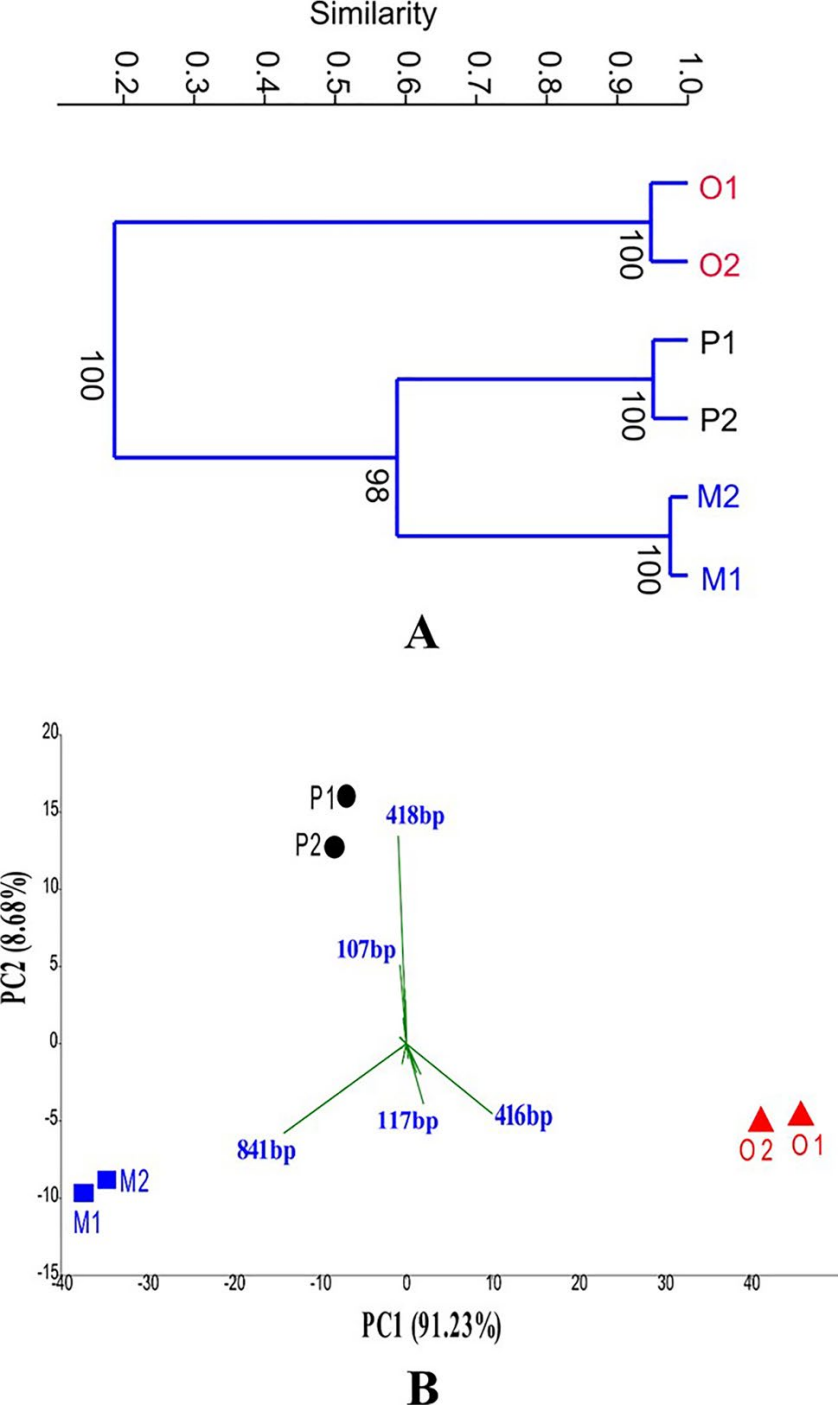
(A) Cluster analysis of the 16S rRNA gene-based T-RFLP electropherograms of the duplicate enrichment cultures at the 5th week by Bray–Curtis algorithm. (B) Principal component analysis 16S rRNA gene-based T-RFLP electropherograms. M1 and M2, *m*-xylene degrading enrichments; P1 and P2, *p*-xylene-degrading enrichments; O1 and O2, *o*-xylene-degrading enrichments

The Bray–Curtis similarity-based cluster analysis of the T-RFLP profiles showed intercorrelation-based hierarchical clustering of beta diversity of enrichment samples, considering T-RF fragment abundances. The UniFrac tree showed that the structure of the bacterial community in the *o*-xylene-degrading enrichments was utterly distinct from the other two types of enrichments where it can be observed that *m-* and *p*-xylene-degrading enrichments shared similar microbial communities by clustering together in different subgroups, unlike the *o*-xylene-degrading enrichments (Fig. 3, panel A). Therefore, it is worth mentioning that *o*-xylene-degrading enrichment harbored the most distinct bacterial community. Nevertheless, it was also noticeable that the duplicate enrichments had mostly similar community compositions.

PCA analysis based on 16 s rDNA T-RFLP profiles revealed the remarkable differences between bacterial community structures of *m*-, *p*-, and *o*-xylene-degrading enrichment samples. PCA plot pointed out that the replicate samples clustered together in all the cases and showed visible distance in clustering between *m-*, *p-*, and *o*-xylene-degrading enrichment samples from each other. However, PCA showed that the *o*-xylene-degrading enrichment samples were apparently more distinct microbiologically. In the formation of clusters, the following T-RFs played a significant role: for *o*-xylene 117 bp and 416 bp, for *m*-xylene the 841-bp-long T-RF fragment, while for *p*-xylene-degrading enrichments 107-bp and 418-bp T-RFs (Fig. 3, panel B, green vectors).

Presumably, these T-RFs denote bacteria capable of aerobic degradation of xylene isomers as carbon source and became a dominant member of the community. These results confirmed that the replicates are parallel in nature and confer a similar bacterial community. Positive correlations were shown between the first and second components of PC (PC1 = 91.23%, PC2 = 8.68%, respectively).

Based on the T-RFLP results, enrichment samples, namely, M1, P1, and O1, were selected for Illumina 16S rRNA gene amplicon sequencing. To evaluate differences in the alpha diversity of OTUs, rarefaction curve was created for each individual sample showing the number of observed OTUs, defined at a 97% sequence similarity cut-off in Mothur (Schloss et al. 2009) relative to the number of total identified bacterial rRNA gene sequences (Fig. S1). In general, all the enrichments were rich and diverse. The graph demonstrated that *m*-xylene-degrading microcosm was the enrichment having higher OTU-based richness, whereas *p*-xylene-degrading enrichment showed much less diversity. All of the samples showed OTU-based saturation around 100–120 OTUs.

**Fig. 4:**
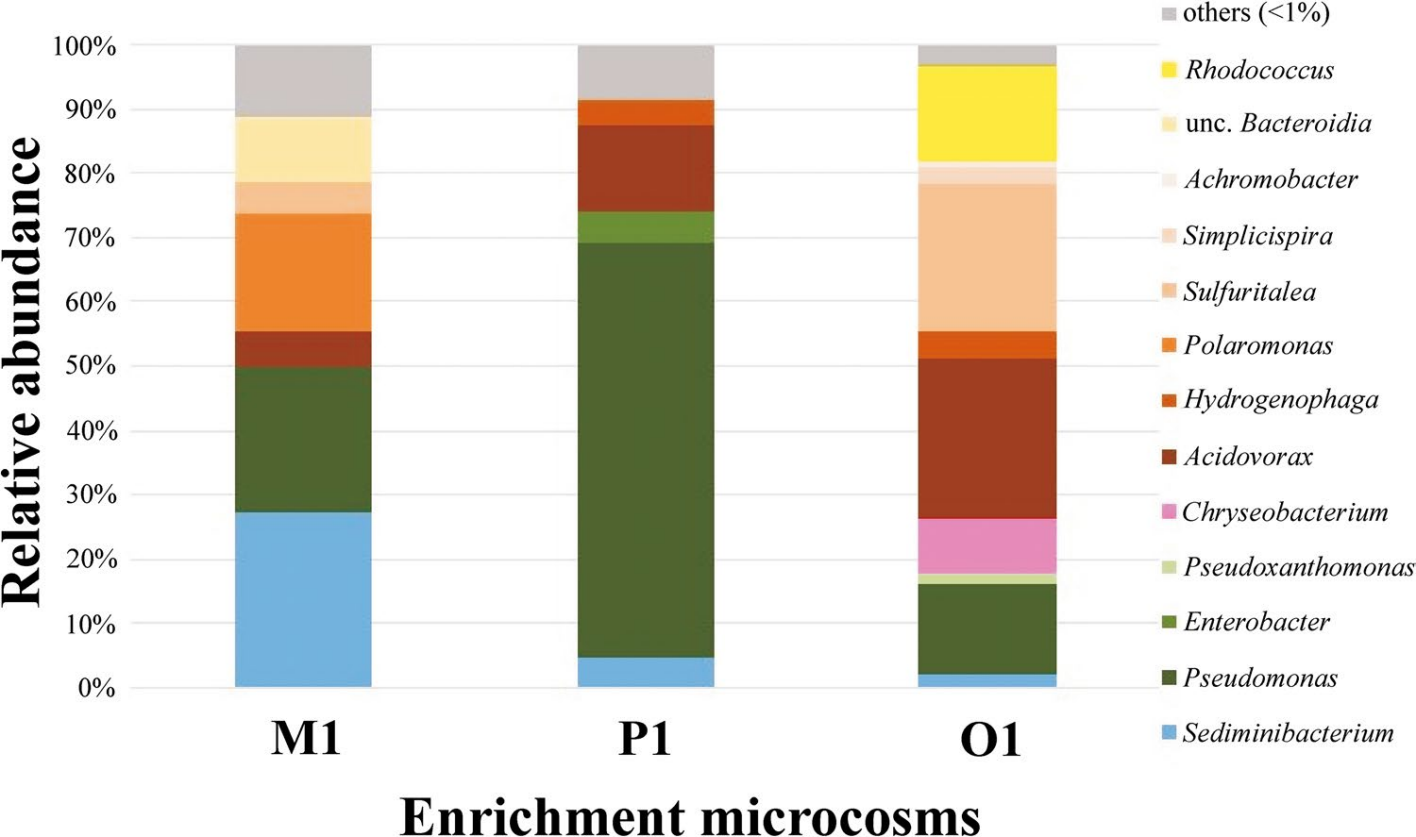
Genus-level bacterial community structure of enrichments M1, P1, and O1 as revealed by Illumina paired-end 16S rRNA gene amplicon sequencing. Only taxa contributing more than 1% abundance were depicted

Finally, a closer, in-depth look at the individual enrichments at class, order, and genus level helped us in understanding the community diversity and composition. At the class level, members of the class *Gammaproteobacteria* dominated the bacterial community in the majority of the enrichments. Enrichment M1 showed the utmost order-based diversity. Both M1 and P1 enrichment communities were dominated by *Gammaproteobacteria* and *Bacteroidia*, and the exception was the O1 enrichment sample where *Actinobacteria* showed more than 15% relative abundance in the community, which was making the community different from the other two enrichments (Fig. 4). Order-based analysis revealed that enrichments were harboring primarily genera belonging to orders *Betaproteobacteriales*, *Pseudomonadales*, *Chitinophagales*, *Corynebacteriales*, and *Bacteroidia*.

The bacterial community of the *m*-xylene-degrading enrichment (M1) was dominated by members of the genera *Sediminibacterium* (27.1%), *Pseudomonas* (22.8%), and *Polaromonas* (18.4%). Whereas in *p*-xylene-degrading enrichment (P1), members of the genus *Pseudomonas* overwhelmingly dominated the community by showing 64% relative abundance (Fig. 4). Besides, members of some other genera like *Acidovorax* (13.2%), *Enterobacter* (5.1%), *Sediminibacterium* (4.6%), and *Hydrogenophaga* (3.9%) were detectable with prominent abundance as well. However, the *o*-xylene-degrading enrichment (O1) showed an altogether different community structure. Though *Pseudomonas*-related bacteria were present in the community but only with 14% abundance. *Acidovorax* (24.9%) was the most dominant genus along with *Sulfuritalea* (22.8%), *Rhodococcus* (14.6%), *Chryseobacterium* (8.4%), and *Hydrogenophaga* (4.4%), making it the most different enrichment based on community diversity in comparison to the two enrichments.

Several among these bacterial genera, such as *Acidovorax*, *Enterobacter*, *Pseudomonas*, *Rhodococcus*, *Sulfuritalea*, *Simplicispira*, *Hydrogenophaga*, and *Polaromonas*, have been either found to be present in petroleum-contaminated environments or known to be effective aromatic hydrocarbon degraders (Nishino et al. 2000; Margesin et al. 2003; Jeon et al. 2004; Mattes et al. 2008; Seo et al. 2009; Prince et al. 2010; Varjani and Upasani 2016; Sarkar et al. 2017; Sperfeld et al. 2018; Xu et al. 2020).

Analysis of the Illumina data showed that *m*- and *p*-xylene-degrading enrichments were primarily predominated by members of the genus *Pseudomonas*, marginalizing the growth of other genera. The dominant role of *Pseudomonas*-related bacteria in aromatic hydrocarbon degradation under strictly aerobic conditions is well known. Thus, these organisms were widely investigated as model organisms to study aerobic BTEX degradation. The *p*-xylene-degrading enrichment, which had a high abundance of sequence reads affiliated with genera *Pseudomonas* and *Acidovorax*, enabled us to assume that these bacteria had played a role in the aerobic degradation of *p*-xylene. Members of the genera *Enterobacter* and *Sediminibacterium* (Poi et al. 2018) are frequently observed in petroleum hydrocarbon-contaminated environments, but their role in hydrocarbon degradation is yet not clear (Kaplan and Kitts 2004; Aburto et al. 2009; Singleton et al. 2018). Another major group was *Hydrogenophaga* present in all of the enrichments, which have been selected to study further to reveal their role in the enrichment community. Likewise, *m*-xylene-degrading enrichment was also dominated with *Pseudomonas, Sediminibacterium*, and *Acidovorax*, making the diversity of the enrichment similar to the *p*-xylene-degrading enrichment. The only difference was the introduction of the genus *Polaromonas*, which is reported to be a genus with potential BTEX degraders (Mattes et al. 2008). Contrarily, the *o*-xylene-degrading bacterial community was considerably different due to the presence of genus *Sulfuritalea* and *Rhodococcus* along with *Pseudomonas* and *Acidovorax* as the major players in the community. Moreover, the presence of *Chryseobacterium*, *Simplicispira*, *Pseudoxanthomonas*, and *Achromobacter* in noticeable fractions makes it an interesting and quite different community compared to the other enrichments. Members of the genus *Sulfuritalea* (Sperfeld et al. 2018), *Chryseobacterium* (Guo et al. 2008), *Simplicispira* (Prince et al. 2010), *Pseudoxanthomonas* (Choi et al. 2013), and *Achromobacter* (Guo et al. 2008) were reported to be present in BTEX enrichments and probably took part in the degradation of BTEX compounds as a carbon source under aerobic conditions. In addition to this, *Rhodococcus* was the only dominant genus that was not witnessed in other enrichments, assuming it as the leading player for *o*-xylene degradation (Di Canito et al. 2018). Subsequently, this hypothesis was proven in the BTEX degradation experiment as the isolated *Rhodococcus* strain could effectively degrade *o*-xylene (see below).

Simultaneously, OTU-based bacterial community composition comparison of enrichments using Venn diagrams demonstrated that the *m*-xylene-degrading enrichment was the most diverse community consisting of 57 unique OTUs while *p*- and *o*-xylene-degrading enrichments had 46 and 45 unique OTUs, respectively. The Venn diagram for the bacterial communities of the three enrichments based on NGS data is presented in Fig. S2. The data also showed that the *m*- and *p*-xylene-degrading enrichment shared the highest similarity; in contrast, *o*- and *p*-xylene-degrading enrichments shared the least number of OTUs (only 4). Interestingly, these four shared OTUs were among the least abundant population of sample O1. Additionally, OTUs representing *Rhodococcus* and *Simplicispira* were the exclusive abundant genera in *o*-xylene-degrading enrichment. These results might explain the *o*-xylene-degrading enrichment community as a unique community. Furthermore, *Hydrogenophaga* was one of the abundant overlapping genera present in all of the enrichments in different proportions.

### Isolates and their BTEX degradation capability

Following classical microbiological techniques, bacterial strains were isolated on R2A agar plates from enrichments that have also been analyzed by Illumina 16S rRNA gene amplicon sequencing. Based on different colony morphology and growth pattern total number of 21 strains have been isolated, among which six isolates originated from *m*-xylene-degrading enrichment, eight isolates from *p*-xylene-degrading enrichment, and seven isolates from *o*-xylene-degrading enrichment (Table 1).

**Table 1:** 16S rRNA gene-based identification of bacterial strains isolated from the enrichments

Strain no	Closest relative (type strain)	Length of 16S rDNA analyzed (bp)	Similarity (%)	Subfamily I.2.C *C23O* gene
***m*-xylene degrading enrichment M1**				
D2M1	*Acidovorax delafieldii* ATCC 17,505	1376	100	+
MT3	*Pseudacidovorax intermedius* DSM 21,352	1420	99.93	+
D3M1	*Polaromonas eurypsychrophila* CGMCC:1.15322	1425	99.93	+
D3M2	*Lysobacter sediminicola* JCM 18,205	1445	98.47	−
MW1	*Pseudomonas fluorescens* ATCC 13,525	1432	99.93	+
MT4	*Achromobacter xylosoxidans* ATCC 27,061	1416	99.93	−
***p*-xylene degrading enrichment P1**				
D2P1	*Hydrogenophaga taeniospiralis* CCUG 15,921	1402	99.05	+ *
D2P3	*Hydrogenophaga taeniospiralis* CCUG 15,921	1418	99.15	+
D2P5	*Acidovorax delafieldii* ATCC 17,505	1428	99.93	+
D3P2	*Mycolicibacterium vanbaalenii* DSM 7251	1410	100	−
P1W2	*Pseudomonas putida* ATCC 12,633	1428	99.86	+
P1W3	*Pseudomonas veronii* ATCC 700,272	1427	100	−
PT4	*Pseudomonas putida* ATCC 12,633	1441	99.86	+
PW1	*Enterobacter roggenkampii* DSM 16,690	1438	99.93	−
***o*-xylene degrading enrichment O1**				
D201	*Pseudomonas chlororaphis* subsp. *piscium* DSM 21,509	1402	100	−
D202	*Achromobacter xylosoxidans* ATCC 27,061	1419	99.93	−
D203	*Microbacterium paraoxydans* DSM 15,019	1412	99.93	−
D204	*Rhodococcus imtechensis* DSM 45,091	1409	99.5	−
0W2	*Pseudomonas chlororaphis* subsp. *piscium* DSM 21,509	1432	100	−
OW3	*Pseudomonas chlororaphis* subsp. *piscium* DSM 21,509	1402	100	−
OY1	*Pseudomonas chlororaphis* subsp. *aureofaciens* ATCC 13,985	1419	99.93	−

^*^Mixed sequence electropherogram was obtained after Sanger sequencing of the I.2.C C23O amplicon, assuming the presence of more than one I.2.C *C23O* genotype in the genome

Strains isolated from the *m*-xylene-degrading enrichment (M1) belonged to the genera of *Acidovorax*, *Pseudacidovorax*, *Polaromonas*, *Lysobacter*, *Pseudomonas*, and *Achromobacter*. Amid those strains, *Acidovorax*, *Pseudacidovorax*, *Polaromonas*, and *Pseudomonas* possessed subfamily I.2.C-type *C23O* gene, which was sequenced further to study functional gene diversity in the isolated community members. Among those strains, *Polaromonas* and *Pseudacidovorax* were selected for further degradation study because of limited available knowledge on their role as potential xylene degraders. Unfortunately, the *Polaromonas* strain was lost during subculturing. Hence, BTEX degradation ability of *Pseudacidovorax* was only checked and observed that it could degrade 5 mg/L concentration of benzene and ethylbenzene in approximately 48 h (data not shown). In contrast, it was unable to degrade toluene and any isomer of xylene.

**Fig. 5:**
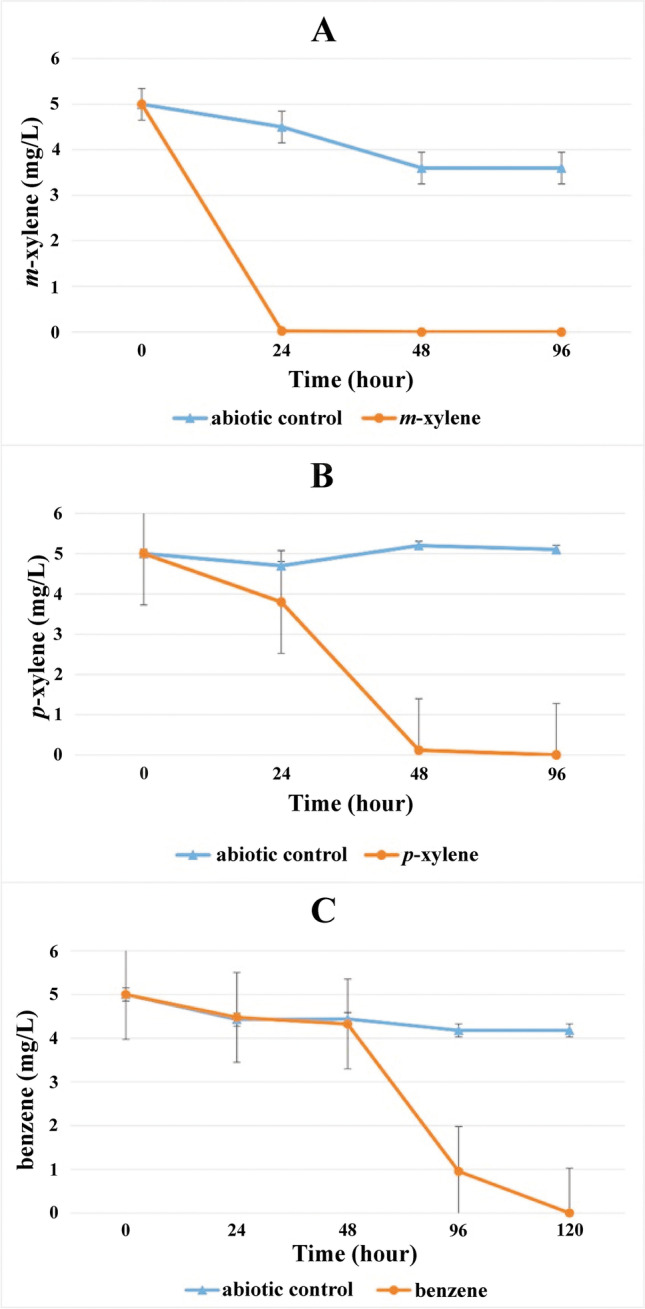
Aerobic degradation of (A) *m*-xylene, (B) *p*-xylene, and (C) benzene by *Hydrogenophaga* sp. strain D2P1. Concentrations were determined by GC–MS analysis as described in the main text. The averages of triplicate experiments ± standard errors of the means, indicated by error bars, are shown

**Fig. 6:**
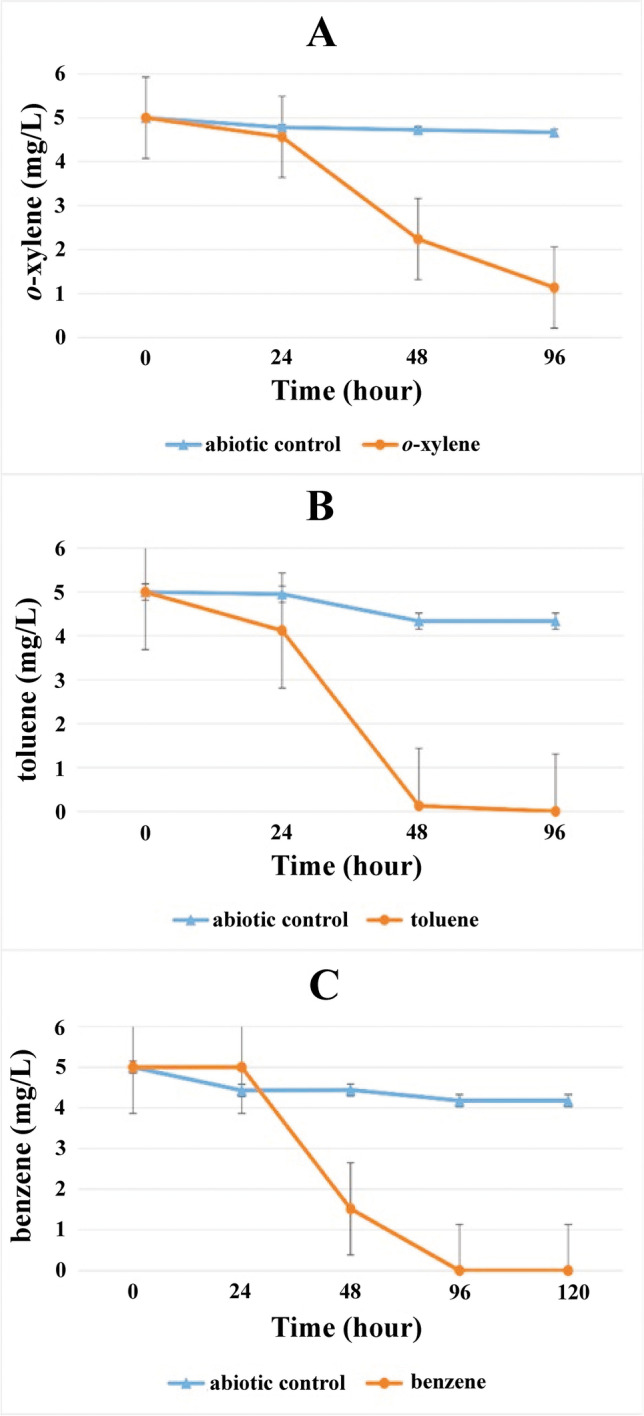
Aerobic degradation of (A) *o*-xylene, (B) toluene, and (C) benzene by *Hydrogenophaga* sp. strain D2P3. Concentrations were determined by GC–MS analysis as described in the main text. The averages of triplicate experiments ± standard errors of the means, indicated by error bars, are shown

Overall, eight strains were isolated from the *p*-xylene-degrading enrichment (P1), which were members of the genera *Hydrogenophaga*, *Acidovorax*, *Mycolicibacterium*, *Pseudomonas*, and *Enterobacter*. The screening of subfamily I.2.C-type *C23O* gene showed that *Hydrogenophaga*, *Acidovorax*, and *Pseudomonas* strains harbored such a gene. Among these strains, two *Hydrogenophaga* strains (sharing identical 16S rRNA genes) showed limited similarity with their closest relative. The presence of subfamily I.2.C-type *C23O* gene made it a potential candidate of interest as information about their biodegradation potential is not so widely available, also not yet considered as a credible candidate for bioremediation. Hence, the biodegradation ability of both *Hydrogenophaga* strains D2P1 and D2P3 (both showing 99.1% similarity with type strain *Hydrogenophaga taeniospiralis* CCUG 15921^ T^) were studied extensively. Surprisingly, although these two strains belonged to the same species they showed different degradation potential towards different BTEX compounds. In the case of *Hydrogenophaga* sp. strain D2P1 degradability for *m-*, *p*-xylene and benzene was shown earlier (Banerjee et al. 2021). During the present study it was observed that strain D2P1 was capable of degrading 5 mg/L concentration of *m*-xylene within 24 h and *p*-xylene within approximately 48 h of incubation at 28 °C and degradation of same concentration of benzene within around 120 h under similar conditions (Fig. 5). In contrast, *Hydrogenophaga* sp. strain D2P3 showed utilization of completely different BTEX compounds as carbon source. Strain D2P3 was able to degrade 5 mg/L concentration of toluene around 48 h, benzene within 96 h, and *o*-xylene in more than 96-h incubation at 28 °C (Fig. 6).

The lowest diversity of isolates was observable in case of the *o*-xylene-degrading enrichment O1. The seven isolates obtained from this enrichment belonged to four genera. Besides, none of them harbored subfamily I.2.C-type *C23O* gene. Although the *o*-xylene degradation ability of *Rhodococcus* (Charniauskaya et al. 2018) is well known still, the degradation potential of the isolated *Rhodococcus imtechensis* strain D2O4 was assessed, which led us to the observation that it could degrade *o*-xylene, toluene, and benzene (data are not shown), which explains its presence in the *o*-xylene-degrading enrichment community as a key player of *o*-xylene utilization. Alongwith that two strains belonging to genus *Pseudomonas*, *Pseudomonas chlororaphis* subsp. *piscium* and *Pseudomonas chlororaphis* subsp. *aureofaciens* were also investigated to assign their role in the *o*-xylene-degradating community, but unfortunately they showed inability to degrade xylenes as carbon source.

### C﻿﻿omparative whole-genome analysis of two *Hydrogenophaga* sp. strains D2P1^T^ and D2P3, with particular emphasis on aromatic hydrocarbon-degrading ability

**Fig. 7:**
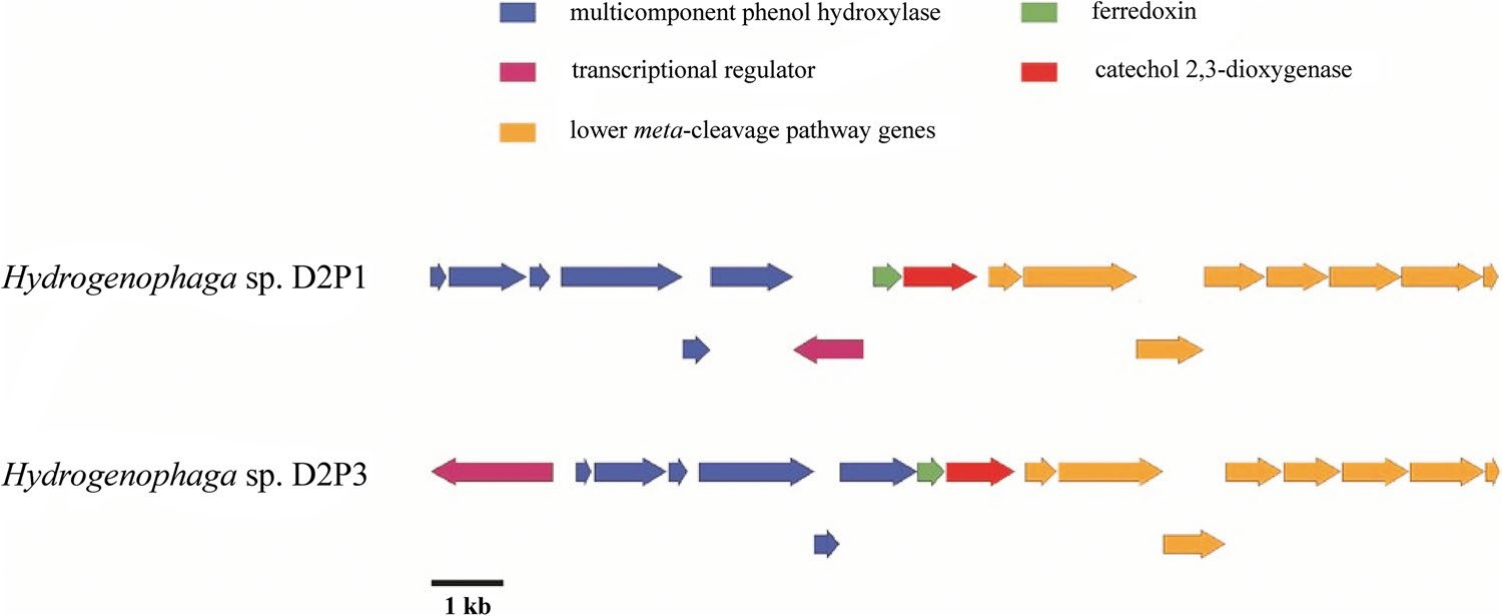
Physical map of the phenol degradation gene clusters of *Hydrogenophaga* sp. strains D2P1^T^ and D2P3

Among the isolates, the two *Hydrogenophaga* strains, D2P1 and D2P3, were the most interesting for us, since they were able to degrade at least one of the xylene isomers and possessed subfamily I.2.C-type *C23O* genes. The two strains shared identical 16S rRNA genes, but Sanger sequencing revealed that they encode entirely different subfamily I.2.C-type *C23O* genotypes. Moreover, based on the Sanger sequencing result, it could be assumed that strain D2P1 harbors more than one genotype of the corresponding *C23O* gene, since mixed sequencing electrophoretogram was obtained. Based on the 16S rRNA gene similarity, strains D2P1 and D2P3 were closely related to *H. taeniospiralis* (~ 99.1% homology), and they shared identical 16S rRNA gene sequence with *Hydrogenophaga* sp. strain Rs71, which was isolated by Fahy et al. (2006) earlier as a benzene-degrading bacterium. Moreover, it was shown that strain Rs71 was able to degrade toluene, *m*-, and *p*-xylene as well (Fahy et al. 2008) similarly to strain D2P1. By the time of writing, strain D2P1 was validly described as type strain of the new species *Hydrogenophaga aromaticivorans*, and the analysis of the whole genome of strain D2P1^T^ revealed that it has three different subfamily I.2.C-type *C23O* genes (Banerjee et al. 2021). Besides, a large gene cluster (~ 28 kbp) was identified, which encoded all of the genes (e.g., xylene monooxygenase and benzoate 1,2-dioxygenase) required for the transformation of *p*- and *m*-xylene to 3- and 4-methylcatechol, respectively (Banerjee et al. 2021). This cluster contained one of the subfamily I.2.C-type *C23O* genes (locus tag F3K02_21385), and it was identified during this study as part of a genomic island, based on the SIGI-HMM algorithm of IslandViewer 4. Since strain D2P3 was not able to utilize *p*- and *m*-xylene, but could use *o*-xylene as sole source of carbon and energy, we sequenced its whole genome as well. Subsequently, the two genomes were aligned to each other and analyzed. The dDDH value between strain D2P1^T^ and D2P3 was 79.7%, while the OrthoANI value was 97.6%, which clearly indicated that they belong to the same genomic species. Besides, they had highly similar genome size (5.63 and 5.80 Mb, respectively). Alignment of the genome sequences revealed that strain D2P3 lacks the xylene-degradation gene cluster, which was observable in case of strain D2P1^T^. On the other hand, both strains harbor a phenol degradation gene cluster, encoding a multicomponent phenol hydroxylase (mPH) together with a complete *meta*-cleavage pathway. However, these gene clusters are different in structure, and the corresponding genes show only ~ 80–90% sequence similarity to each other (Fig. 7). Still, this difference can be the key to understand the different xylene-degrading ability of strains D2P1^T^ and D2P3. It was observed in case of *Pseudomonas stutzeri* strain OX1, which is a prominent toluene and *o*-xylene-degrading bacterium, that a phenol degradation gene cluster plays crucial role in its *o*-xylene-degrading ability. The structure of this phenol degradation operon shows similarity to that of was observed in strain D2P3. Both operons are regulated by a σ^54^-interacting transcriptional regulator and the organization of the mPH and lower *meta*-cleavage genes is similar (at least at the known parts of the operon in case of *P. stutzeri* strain OX1). The C23O enzyme coded in this operon of *P. stutzeri* strain OX1 can cleave 3,4-dimethylcatechol, but cannot cleave 3,5- and 3,6-dimethylcatechols. Consequently, strain OX1 can degrade *o*-xylene, but cannot grow on *m*- and *p*-xylenes (Arenghi et al. 2001). A similar scenario can be assumed in the case of *Hydrogenophaga* sp. strain D2P3. Due to the presence of a single transcriptional regulator it can also be speculated that the whole phenol-degradation gene cluster of this strain is transcribed as an extraordinarily large operon, similarly as in *Pseudomonas* sp. strain CF600 (Shingler, 1996). However, it is still a question how the *o*-xylene is converted into 3,4-dimethylphenol, since toluene-*o*-xylene monooxygenase was not found in the genome of strain D2P3. One possible explanation is that the mPH is responsible for both the hydroxylation of *o*-xylene and the subsequent hydroxylation of 3,4-dimethylphenol to 3,4-dimethylcatechol. Nevertheless, transcriptomic analysis will be necessary to answer this question. In the case of strain D2P1^T^ a LysR-type transcriptional regulator gene was found wedged between the mPH and the ferredoxin gene (Fig. 7). Besides, no σ^54^-interacting transcriptional regulator gene was found upstream of the mPH. Instead, an *orf* encoding an IS5 family transposase was found in the corresponding position, hinting at the possibility that this gene cluster was acquired through horizontal gene transfer (HGT) by strain D2P1^T^. Moreover, it can also be speculated that this gene cluster functions only partially, since the mPH lacks its own transcriptional regulator.

## Conclusion

Based on all above it can be concluded that the microbial community at the Siklos BTEX-contaminated site of Hungary has the metabolic potential to aerobically degrade all isomers of xylene. Polyphasic analysis of the enrichments revealed that distinctly different bacterial communities played role in the degradation of the different xylene isomers. Still, members of the genera *Pseudomonas* and *Acidovorax* were abundant community members in all of the enrichments, while bacteria belonging to the genera *Rhododoccus* and *Chryseobacterium* were key players only in the *o*-xylene-degrading enrichment cultures. It was confirmed by the results that members of the genus *Hydrogenophaga*, harboring subfamily I.2.C-type *C23O* genes, can be prominent xylene-degraders, and some strains can even acquire *o*-xylene-degrading ability as well. In connection with this, the whole-genome analysis of two *H. aromaticivorans* strains revealed that different subpopulations of the same species with different xylene-degrading capabilities may coexist in the same environment. Our findings also shed light on the fact that even three or more subfamily I.2.C-type *C23O* genotypes can be linked to one single species of the degrader community. Consequently, the high diversity of subfamily I.2.C-type *C23O* genes does not necessarily mean high degrader diversity in a contaminated environment.

## Supplementary Information

Below is the link to the electronic supplementary material.

Supplementary file1 (PDF 557 KB)

## Data Availability

Whole-genome sequence data are available under the BioPoject accession number PRJNA565673. 16S rRNA gene amplicon sequencing data are available under the BioProject accession number PRJNA704261. Other datasets used and analyzed during the current study are available from the corresponding author on reasonable request.
