# Supplementary material for: Evaluating the aerobic xylene-degrading potential of the intrinsic microbial community of a legacy BTEX-contaminated aquifer by enrichment culturing coupled with multi-omics analysis: uncovering the role of Hydrogenophaga strains in xylene degradation
Source: Environ Sci Pollut Res Int. 2022 Jan 6;29(19):28431–45. doi: 10.1007/s11356-021-18300-w (PMC8993774; doi:10.1007/s11356-021-18300-w)
Supplement: Supplementary file 1 — Supplementary file1 (PDF 557 KB) [file 11356_2021_18300_MOESM1_ESM.pdf]

Evaluating the aerobic xylene-degrading potential of the intrinsic microbial community of a legacy BTEX-contaminated aquifer by enrichment culturing coupled with multi-omics analysis: uncovering the role of *Hydrogenophaga* strains in xylene degradation

Sinchan Banerjee[1], Anna Bedics[1], Péter Harkai[2], Balázs Kriszt[2], Nagaraju Alpula[1,3] András Táncsics[1]

[1]Department of Molecular Ecology, Institute of Aquaculture and Environmental Safety, Hungarian University of Agriculture and Life Sciences, Gödöllő, Hungary

[2]Department of Environmental Safety, Institute of Aquaculture and Environmental Safety, Hungarian University of Agriculture and Life Sciences, Gödöllő, Hungary

[3]Microbial Biotechnology Research Unit, Department of Biotechnology, Kakatiya University, India

Journal: Environmental Science and Pollution Research

Correspondence: András Táncsics, Department of Molecular Ecology, Institute of Aquaculture and Environmental Safety, Hungarian University of Agriculture and Life Sciences, Páter K. u. 1., H-2100 Gödöllő, Hungary. E-mail: tancsics.andras@uni-mate.hu

Table S1:

Year	BTEX compounds (µg/L)					Physicochemical parameters							
	Well code	Xylene	Benzene	Toluene	Ethyl-benzene	Temperature (°C)	pH	DO (mg/L)	NO_3^- (mg/L)	Fe_2^+ (mg/ L)	SO_4^{2-} (mg/L)	CH_4 (mg/L)	Redox potential (mV)†
**2011***	SKV	<2	<0.2	<1	<1	14.2	6.7	3.3	61	<0.02	71	<0.04	369
	**ST2**	**6700**	**340**	**64**	**966**	**14.3**	**8.0**	**0.6**	**2**	**4**	**24**	**1.9**	**148**
**2019**	SKV	<2	<0.2	<1	<1	14.5	6.8	2.1	40	0.13	60	<0.04	14
	ST2	**2760**	**227**	**15**	**862**	**14.2**	**7.6**	**1.2**	**<5**	**8.2**	**<30**	**2.19**	-42

†measured with a standard hydrogen reference electrode.
*Data source: Táncsics et al 2012

Table S2: Alpha diversity index of enrichment samples (M= *m*-xylene-degrading enrichments, P= *p*-xylene-degrading enrichments , O= *o*-xylene-degrading enrichments)

	**M1**	**M2**	**P1**	**P2**	**O1**	**O2**
**Simpson_1-D**	0.4684	0.5013	0.7699	0.7507	0.7669	0.7841
**Shannon_H**	1.22	1.297	1.818	1.757	1.999	2.065
**Chao-1**	12	12	10	10	15	15

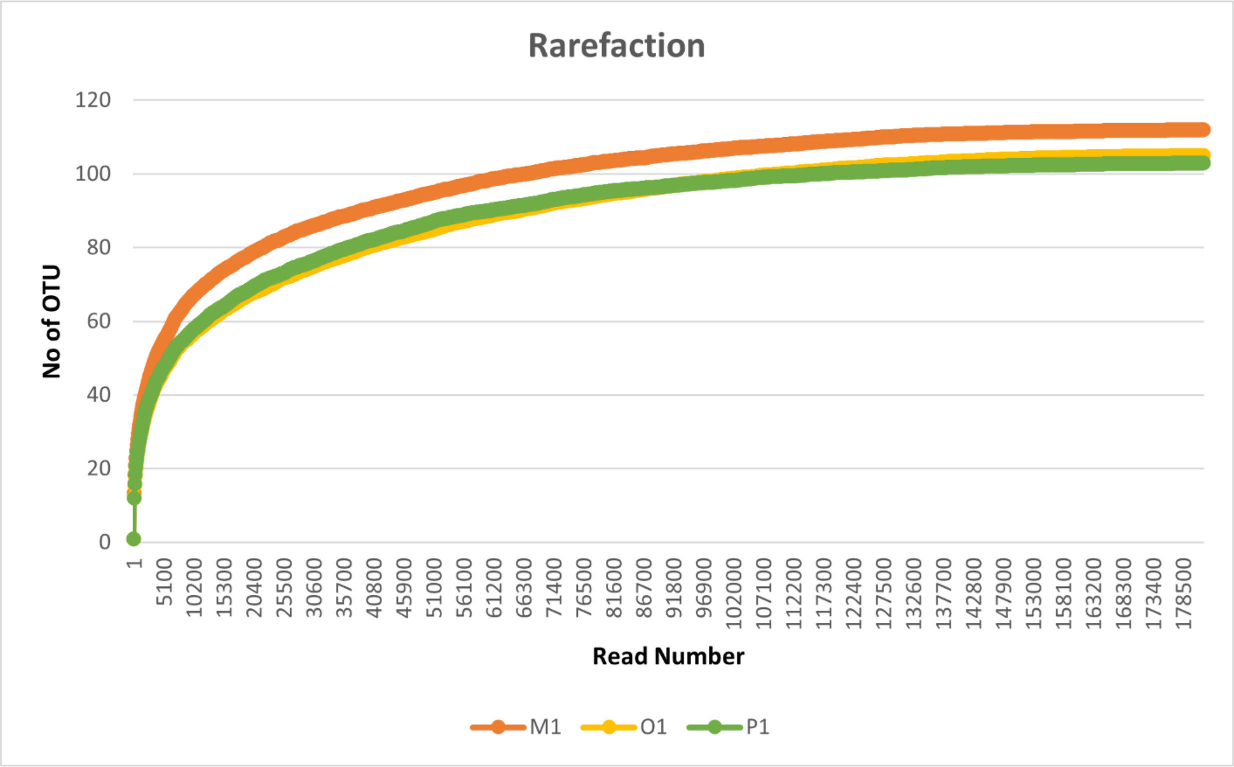

Figure S1: Rarefaction curves were assembled showing the number of OTUs, defined at the 97% sequence similarity cut-off in Mothur, relative to the number of total sequences

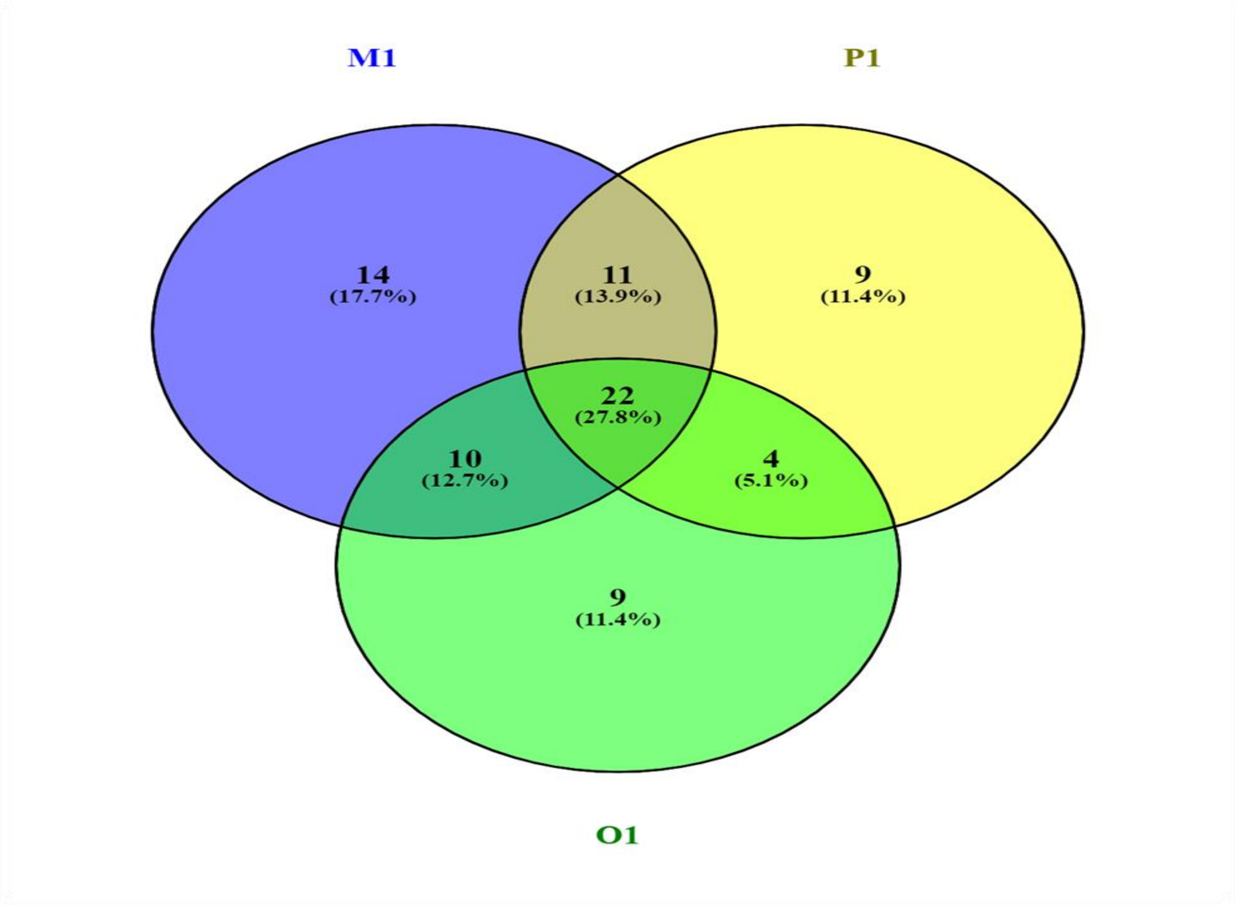

Figure S2: Venn diagram showing shared and unique genera at 97% identity among the three groups M1 (*m*-xylene-degrading enrichment), P1 (*p*-xylene-degrading enrichment) and O1 (*o*-xylene-degrading enrichment). Based on Genus with relative abundance more than 1%.
